# Supplementary material for: Discovery and pharmacophoric characterization of chemokine network inhibitors using phage-display, saturation mutagenesis and computational modelling
Source: Nat Commun. 2023 Sep 16;14:5763. doi: 10.1038/s41467-023-41488-z (PMC10505172; doi:10.1038/s41467-023-41488-z)
Supplement: Supplementary file 4 — Source Data [file 41467_2023_41488_MOESM4_ESM.zip › SourceData/scripts/xattune_20230807170606.tar!/xattune/analysis/attune_ic50/SV_E25_CCL8_HD2_plot.pdf]

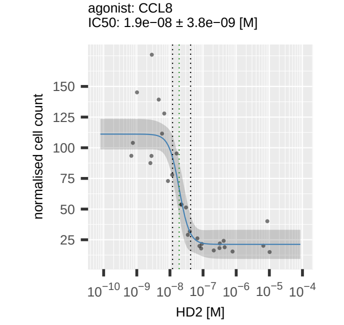

agonist: CCL8
IC50: 1.9e−08 ± 3.8e−09 [M]
normalised cell count
150
125
100
75
50
25
10−10
10−9
10−8
10−7
10−6
10−5
10−4
HD2 [M]
